# Athletes: Fit but Unhealthy?

**DOI:** 10.1186/s40798-016-0048-x

**Published:** 2016-05-26

**Authors:** Philip B. Maffetone, Paul B. Laursen

**Affiliations:** 1 Oracle, AZ USA; 2 Sports Performance Research Institute New Zealand (SPRINZ), Auckland University of Technology, Auckland, New Zealand; 3 High Performance Sport New Zealand, PO Box 302 563, North Harbour, Auckland, 0751 New Zealand

## Abstract

While the words “fit” and “healthy” are often used synonymously in everyday language, the terms have entirely separate meanings. Fitness describes the ability to perform a given exercise task, and health explains a person’s state of well-being, where physiological systems work in harmony. Although we typically view athletes as fit and healthy, they often are not. The global term we place on unhealthy athletes is the overtraining syndrome. In this current opinion, we propose that two primary drivers may contribute to the development of the overtraining syndrome, namely high training intensity and the modern-day highly processed, high glycemic diet. Both factors elicit a sympathetic response through the hypothalamic-pituitary-adrenal axis, in turn driving systemic reactive oxygen species production, inflammation, and a metabolic substrate imbalance towards carbohydrate and away from fat oxidation, manifesting in an array of symptoms often labeled as the overtraining syndrome. Ultimately, these symptoms reveal an unhealthy athlete. We argue that practitioners, scientists, and athletes may work towards health and alleviate overtraining syndrome by lowering training intensity and removing processed and/or high glycemic foods from the diet, which together enhance fat oxidation rates. Athletes should be fit and healthy.

## Key Points

1. Fitness and health can be defined separately: fitness describes the ability to perform a given exercise task, and health explains a person’s state of well-being, where physiological systems work in harmony.
2. Too many athletes are fit but unhealthy.
3. Excess high training intensity or training volume and/or excess consumption of processed/refined dietary carbohydrates can contribute to reduced health in athletes and even impair performance.

## Background

This current opinion piece combines the art and science perspectives of a clinician/coach (PM) and an applied sport scientist/physiologist (PL), both with specializations in endurance performance. Our collaboration arises from a mutual respect for contributions to endurance athlete performance, as well as a shared primary observation—that of fit, but unhealthy recreational and professional/elite athletes.

## Health and Fitness: Separate and Equally Important

The term unhealthy athlete sounds, at first blush, like a paradox. The magazine cover image of an athlete performing her event in all its glory with flexed, lean muscles, bronzed skin, and glowing good looks may be perceived as the pinnacle of health. The internal working state of that athlete, however, may be at arm’s length from genuine health. In actuality, an athlete can be fit but unhealthy (Fig. 1).

**Fig. 1:**
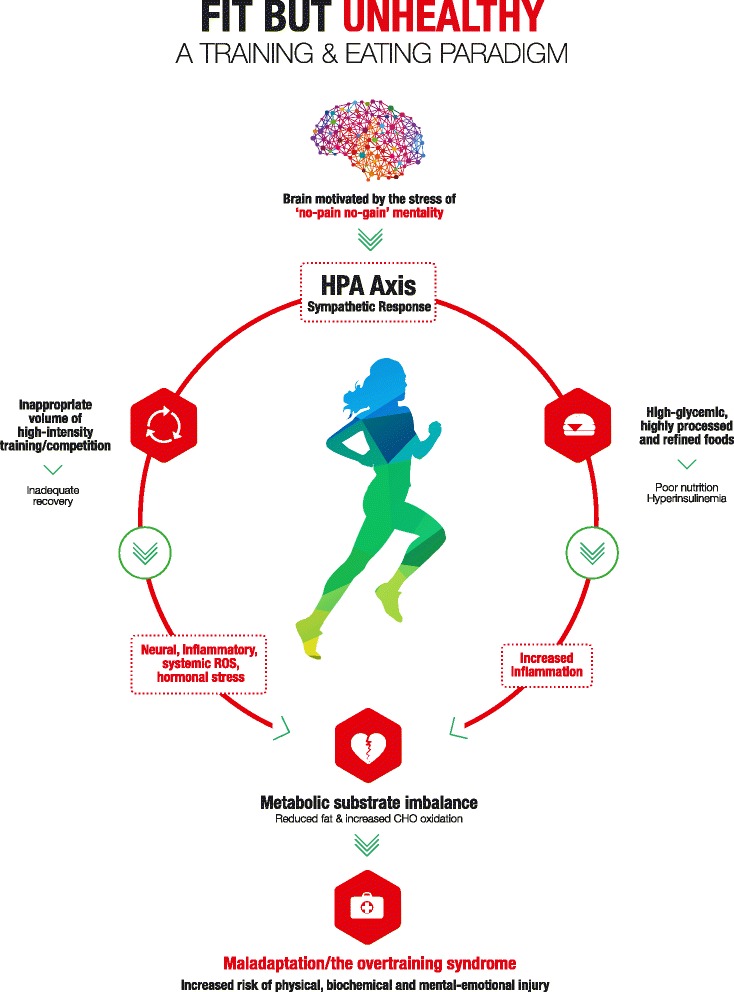
The fit but unhealthy training and eating paradigm

While the terms “health” and “fitness” are often used interchangeably, we offer separate definitions:

- Health: a state of complete, mental, social, and physical well-being, where all bodily systems (nervous, hormonal, immune, digestive, etc.) function in harmony
- Fitness: the quality of being able to perform a specific physical task, which includes exercise and sports

Poor health can be observed in athletes who adhere to sport’s global “no pain, no gain” mentality, who may push themselves beyond a point of appropriate system stress [1, 2]. This includes physical injury (e.g., neuromuscular dysfunction), biochemical injury (e.g., endocrine and immune dysfunction), and/or mental-emotional injury (e.g., depression). Each injury, in turn, could potentially cause other signs and symptoms indicative of poor health. The overreaching label we place on many athletes presenting with various combinations of these injuries is the overtraining syndrome [3]. The mechanisms leading to this condition can vary considerably, across different levels of training history and ability (recreational, non-elite, elite athletes), age, genders, and sports (dependent upon their aerobic and/or anaerobic metabolic requirements). The wide spectrum of various potential injuries is listed in Table 1 [4].

**Table 1:** Some potential physical, biochemical, and mental-emotional signs and symptoms associated with the overtraining syndrome. Adapted from Meeusen et al. [3] and Kreher and Schwartz [4]

Category	Signs and symptoms
Physical	Maximal and submaximal performance decrements Muscle soreness/stiffness/fatigue General persistent fatigue Bradycardia and altered heart rate variability Autonomic imbalance (including cardiac) Disease conditions: asthma, thyroid disease, adrenal disease, diabetes mellitus or insipidus, iron deficiency with or without anemia, celiac sprue, hypertension
Biochemical	Excessive oxidative stress/damage Increased susceptibility to viral, bacterial, and other infections Inflammation Malnutrition Hormone imbalance Reduced muscle glycogen levels
Mental-emotional	Disordered eating Depression Insomnia Disrupted mood/behavior Loss of motivation Reduced mental concentration Anxiety

Important determinants of population health include genetic predispositions, social circumstances, environmental conditions, behavioral patterns (including diet and exercise), and medical care [5]. Fitness factors contributing to the various conditions of the overtraining syndrome may be similar and are certainly multifaceted, interrelated, and complex [3]. Our opinion, based on practical experience and growing support from the scientific community, is that there may be two modifiable primary drivers of these injuries. This includes training intensity and the modern diet. Both appear to conflict with our genetic makeup [1], and both influence an important biochemical characteristic—the oxidation of fat for fuel [6].

## Our Athletic Origins

From the emergence of the human genus, *Homo*, about 2.4 million years ago, our ancestors survived as hunter-gatherers for approximately 84,000 generations. They required large expenditures of energy on a daily basis derived from lipid oxidation that supported the primary activities of walking, slow running, resting, and the occasional sprint, while adapting to a rapidly evolving glucose-based brain [7]. More recently, dramatic advances in technology by the agricultural (350 generations), industrial (7 generations), and digital (2 generations) revolutions have left us with a population that is genetically adapted for the demands of life as a forager in the wild, but living in a high-tech, generally sedentary, overfed, and emotionally stressful twenty-first century world [8]. While the majority of the population today suffers from the problem of inactivity, there is a smaller group, namely athletes, which may be exercising excessively, or at a level inappropriate to maintain optimal health [4].

Our bodies obtain substantial energy from stored body fat, even in the leanest of individuals. Lipid oxidation, the biochemical conversion of stored body fat for aerobic energy, also has a mechanical element, the slow-twitch aerobic muscle fibers [9]. As the name implies, these fibers are enlisted during slower or lower-intensity activities and play an important role in the health of skeletal and joint support, vascularization, and antioxidant activity [9]. These fibers and their lipid-derived fuel supply form a critical component of the aerobic system for endurance, defined here as the ability to train and compete over longer timeframes, with less fatigue and at faster paces [10].

## Diet

Today’s diet is typically high in refined carbohydrate, including various forms of sugar, which many athletes erroneously believe is necessary for better performance [11, 12]. However, a diet high in refined carbohydrate is also high glycemic, which can promote poor health [13, 14]. In the short term, refined carbohydrates, whether in the form of sugar or refined flour, can impair fat oxidation rates [13, 15] and contribute to the production of inflammation and pain [16, 17] and increased reactive oxygen species (ROS) production [14]. Over time, this can create hyperinsulinemia and chronic inflammation, also associated with reduced health [18]. Other dietary factors such as excess alcohol, excess omega-6 lipid consumption, trans fatty acids and others, along with various lifestyle factors including stress and excess exercise, can also contribute to chronic inflammation [14].

## Training Trauma and Inflammation

Associated with the above potential training and dietary problems are a variety of physical, biochemical, and mental-emotional injuries (Table 1). Whether we call these different components of overtraining syndrome or not, it is the hypothalamic-pituitary-adrenal axis and the autonomic nervous system that play key roles, creating sufficient stress to cause system injury on various levels, and ultimately maladaptation [16, 17, 19].

While acute inflammation is a normal response to exercise training and helps athletes recover and adapt, inappropriate training intensity, volume, or both, especially when combined with a high glycemic/refined CHO diet, can result in chronic inflammation and pain [16, 17], increased ROS [14], a compromised nervous system [19], and various symptoms of maladaptation [2]. This may contribute to a wide spectrum of undesirable health outcomes [19] that may vary considerably among athletes and can include plantar fasciitis, tendonitis, or stress-related health impairment as part of the overtraining syndrome and chronic disease risk (Table 1).

## Potential Solutions

Notwithstanding a multitude of other potentially contributing factors to the various injuries outlined, addressing the possible damaging effects of excessive high-intensity training and/or poor diet is an important consideration. While high-intensity training can be important to implement in moderation and at the right time [20] for certain elements of sport performance [21], it does little to enhance fat oxidation rates [22], unlike lower-intensity aerobic training [23]. Likewise, reducing refined carbohydrate content from an athlete’s diet and replacing these calories with healthy/natural carbohydrate and fat over prolonged periods can increase both lipid oxidation rates and various markers of metabolic health [13, 15].

From a practical standpoint, the modification of the diet and/or training programs as indicated can improve health while increasing fitness, potentially through the mechanisms discussed above.

## Conclusions

Physical, biochemical, and mental-emotional injuries are not expected/normal outcomes from endurance sport participation, yet the incidence of these in athletes is alarmingly high. Practitioners, coaches, and athletes should be cognizant of impending health abnormalities during training and consider periods of reduced training intensity and recovery, while emphasizing a natural, unprocessed diet to improve health and cultivate sustainable fitness. For optimal performance, athletes must be fit *and* healthy.
